# Re-Emergence of Yellow Fever in Brazil during 2016–2019: Challenges, Lessons Learned, and Perspectives

**DOI:** 10.3390/v12111233

**Published:** 2020-10-30

**Authors:** Poliana de Oliveira Figueiredo, Ana Gabriella Stoffella-Dutra, Galileu Barbosa Costa, Jaqueline Silva de Oliveira, Carolina Dourado Amaral, Juliane Duarte Santos, Kamila Lorene Soares Rocha, João Pessoa Araújo Júnior, Maurício Lacerda Nogueira, Magno Augusto Zazá Borges, Adriano Pereira Paglia, Angelle Desiree LaBeaud, Jônatas Santos Abrahão, Erna Geessien Kroon, Danilo Bretas de Oliveira, Betânia Paiva Drumond, Giliane de Souza Trindade

**Affiliations:** 1 Laboratório de Vírus, Departamento de Microbiologia, Instituto de Ciências Biológicas, Universidade Federal de Minas Gerais, Belo Horizonte, Minas Gerais 31270-901, Brazil; polianaofigueiredo@yahoo.com.br (P.d.O.F.); jaquelinebmedica@hotmail.com (J.S.d.O.); carolinadamaral@gmail.com (C.D.A.); jonatas.abrahao@gmail.com (J.S.A.); ernagkroon@gmail.com (E.G.K.); betaniadrumond@gmail.com (B.P.D.); 2 Laboratório de Patologia e Biologia Molecular, Instituto Gonçalo Moniz, Oswaldo Cruz Foundation, Rua Waldemar Falcão, 121, Candeal, Salvador Bahia 40296-710, Brazil; 3 Centro Integrado de Pesquisa em Saúde, Faculdade de Medicina, Universidade Federal dos Vales do Jequitinhonha e Mucuri Campus JK, Diamantina, Minas Gerais, Rodovia MGT 367, Km 583, nº 5.000 Alto da Jacuba 39100-000, Brazil; julianeduartesantos@gmail.com (J.D.S.); kamilalsr@yahoo.com.br (K.L.S.R.); danilo.bretas@ufvjm.edu.br (D.B.d.O.); 4 Departamento de Microbiologia e Imunologia, Institute of Biotechnology, Universidade Estadual Paulista Júlio de Mesquita Filho, Rio Claro, São Paulo Avenida 24A, 1515, Bela Vista 13506-900, Brazil; joao.pessoa@unesp.br; 5 Laboratório de Pesquisas em Virologia, Departamento de Doenças Infecciosas e Parasitárias, Faculdade de Medicina de São José do Rio Preto, São José do Rio Preto, São Paulo 15090-000, Brazil; mnogueira@famerp.br; 6 Centro de Ciências Biológicas e da Saúde, Universidade Estadual de Montes Claros, Montes Claros, Minas Gerais, Avenida Prof. Rui Braga, s/n, Vila Mauriceia 39408-354, Brazil; magno.borges@unimontes.br; 7 Laboratório de Ecologia e Conservação, Departamento de Biologia Geral, Instituto de Ciências Biológicas, Universidade Federal de Minas Gerais, Belo Horizonte, Minas Gerais 31270-901, Brazil; apaglia@icb.ufmg.br; 8 Department of Pediatrics, Division of Infectious Diseases, Stanford University School of Medicine, 300 Pasteur Dr Rm G312 MC 5208, Stanford, CA 94305, USA; dlabeaud@stanford.edu

**Keywords:** yellow fever, epidemic, epidemiology, ecology, public health, Brazil

## Abstract

Yellow fever (YF) is a re-emerging viral zoonosis caused by the *Yellow Fever virus* (YFV), affecting humans and non-human primates (NHP). YF is endemic in South America and Africa, being considered a burden for public health worldwide despite the availability of an effective vaccine. Acute infectious disease can progress to severe hemorrhagic conditions and has high rates of morbidity and mortality in endemic countries. In 2016, Brazil started experiencing one of the most significant YF epidemics in its history, with lots of deaths being reported in regions that were previously considered free of the disease. Here, we reviewed the historical aspects of YF in Brazil, the epidemiology of the disease, the challenges that remain in Brazil’s public health context, the main lessons learned from the recent outbreaks, and our perspective for facing future YF epidemics.

## 1. Introduction

*Yellow fever virus* (YFV) is a positive-strand RNA virus that is the prototype of *Flavivirus* genus (*Flaviviridae* family) and is recognized as the etiological agent of Yellow Fever (YF) [1,2]. YF disease is characterized by an acute, febrile, hemorrhagic infectious disease, transmitted by mosquito vectors to human populations and non-human primates (NHP) in South America and Africa [2,3]. In Brazil, YF is considered a disease of compulsory notification, where all suspected cases must be immediately reported to the health authorities [2,4].

YF was responsible for hundreds of thousands of deaths between the 18th century and the beginning of the 20th century, and for recurrent epidemics in endemic regions of Africa and South America [2,5]. YF still remains a public health threat, leading to significant morbidity and mortality rates in the human populations of Africa and South America. A high case fatality rate (CFR) is observed, especially in South America, ranging from 40% to 60% [3,5,6]. The occurrence of rural (savannah cycles) and urban cycles is frequently reported in the old world, in addition to sylvatic cycles [7]. Large YF outbreaks occurred in Angola and the Democratic Republic of Congo during 2015–2016 [5,8], which placed YFV on the top list of arboviral threats by the Centers for Disease Control and Prevention (CDC) Global Disease Detection Operations Center [9]. According to the Pan American Health Organization (PAHO), the South American countries that reported the highest numbers of cases of YF during 1960–2019 were Brazil (3829 cases), Peru (3189 cases), Bolivia (1546 cases) and Colombia (701 cases) [10].

In Brazil, YFV is maintained in nature through enzootic/sylvatic cycles involving non-human primates (NHP) and mosquitoes of the genera *Haemagogus* and *Sabethes* [2,11]. YF has a seasonal pattern of occurrence, with most cases recorded from December to May. However, the occurrence of outbreaks is irregular, and viral transmission can change according to factors such as temperature, rainfall, high density of vectors, amplifying hosts, and low vaccination coverage of the human population [2]. Some factors have contributed to the elimination of the YF urban cycle, transmitted by urban vector *Aedes aegypti*, which include the introduction of vaccination since 1937, the mass immunization in the following decade, along with intense campaigns to eradicate the vector [2]. In this scenario, the last registered urban YF case in the country occurred in 1942, followed by epidemic records related to sylvatic cycles, especially in the Amazon basin [2,11].

However, in 2016, one of the most significant epidemics of sylvatic YF occurred in Brazil, with most cases reported in regions considered free of the disease, or with little YFV circulation [5,11,12]. The YF cases exponentially increased during the 2016–2019 epidemic, highlighting concerns about the risks of YF reurbanization once the YFV outbreak reached the southeast region, the most populous region in the country [5,11]. The risk of reurbanization is sustained by vector plasticity in the Brazilian territory and by large susceptible populations that had no routine vaccination until the recent re-emergence [11,13,14,15].

In this review, we revisited the history of YF in Brazil and the substantial impact for public health since its introduction during the colonization period until the emergence observed in the 2016–2019 epidemic. Eco-epidemiological aspects of the disease, as well as the lessons and challenges from last epidemic period, are also discussed.

## 2. A Brief History of Yellow Fever in Brazil

**Figure 1:**
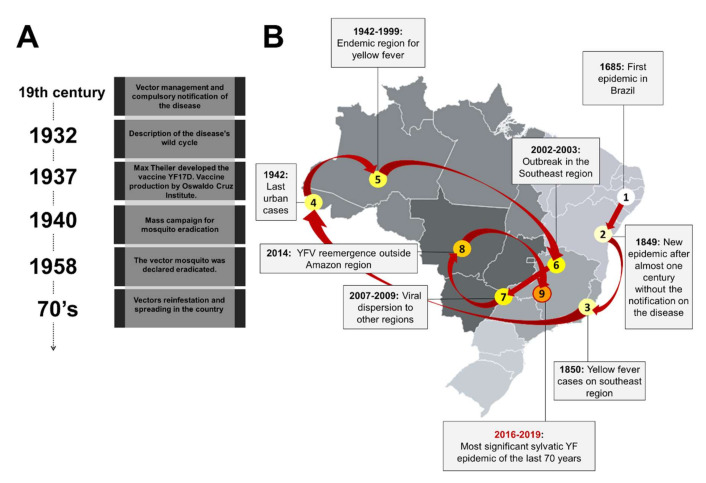
Map of the natural history of Yellow Fever in Brazil. (**A**) The timeline highlights sanitary measures adopted to fight against Yellow Fever in Brazil. (**B**) A map of Brazil showing the detection and distribution of *Yellow Fever virus* (YFV). The red lines connect the events of viral emergence in different regions of the country. The grey scale indicates the regions of Brazil from the lightest to the darkest as follows South, Northeast, Southeast, North, and Midwest.

The YFV and its urban vector *Aedes aegypti* arrived in the Americas, including Brazil, through the slave-trading ships from West Africa during the period of colonization [5,8,16,17]. The first YF epidemic in Brazil was recorded in 1685, in the Northeast region, specifically in Recife and Pernambuco states [16,18] (Figure 1).

In the subsequent years, YFV would hit other port cities in the Northeastern region, causing outbreaks. In the middle of 1850, after almost a century without notifications, YFV reached Rio de Janeiro (Southeastern region), causing more than 4000 deaths. Although the etiology of YF was still unknown and there was no proof of any transmission form, at the beginning of the 19th century several surveillance measures were adopted to fight against YF, including the mandatory notification of the disease and the hygienic and sanitary measures that indirectly contributed to the reduction in *Aedes* populations [16,19] (Figure 1). At the end of the 19th century, Carlos Finlay, a Cuban epidemiologist, proposed that YF was transmitted through mosquito bites [20,21,22]. However, it was not until 1900 that Walter Reed, a pathologist and bacteriologist, and his colleagues proved that YF was caused by a filterable agent and transmitted by the vector *Aedes aegypti* [23,24,25,26].

In the following years, because of a decrease in YF cases, the resources to fight against the disease were decreased, contributing to an urban YF epidemic in Rio de Janeiro in 1928–1929 [19]. In addition, in the 1930s, the sylvatic cycle was documented in the country [23,24,25] together with the discovery of the importance of NHP in the viral maintenance cycle [27].

Max Theiler, a South African doctor, developed a mouse model of YF infection to demonstrate the potential of protection of serum antibodies against YFV. In the subsequent years, other research groups investigated the possibility of attenuating a wild type YFV, aiming to induce protective immunity in humans without causing any disease. As a result, the used wild type YFV was attenuated due to the development of an immunogenic and safe Yellow Fever-17D (YF-17D) vaccine strain [28]. In 1937, the vaccine against YF was produced by the Oswaldo Cruz Institute, today called Bio Manguinhos/Fiocruz [16,17,18,19,25,29]. In addition, by 1940 mass campaigns to eradicate the urban vector *Aedes aegypti* had begun; however, sporadic cases still occurred in several states, with the last urban case reported in Acre in 1942 [16,25]. In 1958, the PAHO officially declared that *A. aegypti* was eradicated in Brazil [16]; nevertheless, in the 1970s, the collapse of the continental program to combat vector mosquitoes led by PAHO culminated with the urban reinfestation of *A. aegypti*, and its spread to several Brazilian regions by the end of this decade [30,31,32,33].

The last two decades have witnessed the expansion of YFV circulation area in the country, where human cases and NHP epizootics were registered beyond the endemic Amazon region [34]. YFV spread to the East and South regions could be seen during 2002–2003, with cases registered in Minas Gerais and Rio Grande do Sul, and between 2007 and 2009, with confirmed cases in the North and Midwest regions, in addition to São Paulo, Paraná, and Rio Grande do Sul states [34,35,36]. The change in the spatial distribution of YF cases was even more evident during 2007–2009, with the confirmation of more than 100 YF cases in the South and Southeast regions, with a lethality rate of 51% [32,34,35] (Figure 1).

In 2014, YFV re-emerged in the Midwest region, in areas of Cerrado biome [11,34,37]. In the monitoring period of 2014–2015, based on the seasonality pattern, the occurrence of cases was mainly concentrated in Goiás and Mato Grosso do Sul states, and during 2015–2016 the cases were mostly concentrated in the Midwest region [12,37]. In a historical analysis, from 1980 to 2015, the period that precedes the most recent sylvatic epidemics, 789 YF human cases were registered in Brazil. In this 36-year interval, the outbreaks had an irregular pattern of annual incidence, with some periods of viral re-emergence [37]. At the end of 2016, the most significant sylvatic YF epidemic of the last 70 years began, affecting mainly the Southeastern region of Brazil [11,12,34,38].

## 3. Challenges and Lessons Learned

**Figure 2:**
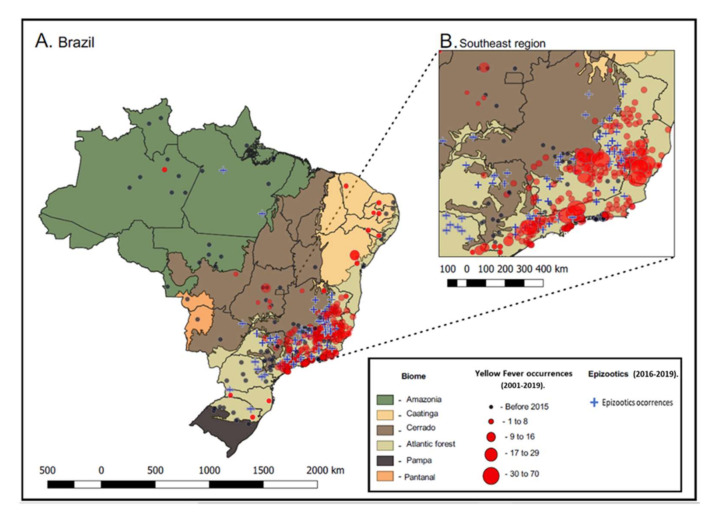
Spatial distribution of Yellow Fever (YF) cases in Brazil during 2001–2019. The map shows a correlation between human confirmed cases of YF and biomes (**A**), and confirmed epizootics in the Southeast region (**B**) during the 2016–2019 outbreak. The map was created using the Quantum GIS (QGIS) software.

The extensive re-emergence of YF in Brazil started in late 2016, and, according to data from the Ministry of Health, 2237 human cases of YF and 759 deaths were recorded between December 2016 and June 2019 [12,39] (Figure 2).

The epicenter of epidemic was the Southeastern region of Brazil, specifically Minas Gerais and São Paulo states [11,12,40,41,42]. This outbreak was 2.8 times greater than what was recorded in the past 36 years [12,38]. In contrast to previous outbreaks concentrated in the Amazon and Central West regions, this outbreak was centered in the Southeastern region of Brazil, covering the Cerrado biome towards the region originally covered by the Atlantic Forest [11].

**Figure 3:**
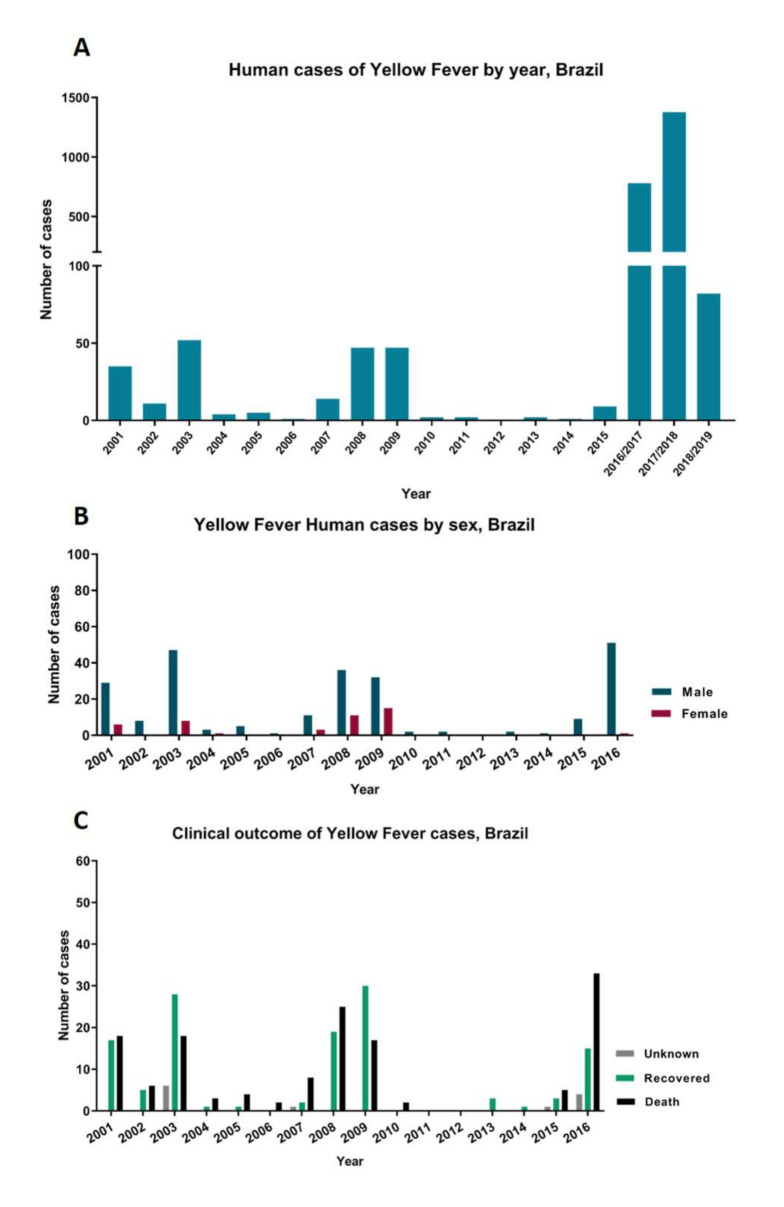
Distribution of human cases of YF in Brazil. (**A**) Distribution of confirmed human cases per year in Brazil for the monitoring period of (July to June) 2001–2019. (**B**) Distribution of YF human cases according to gender in Brazil during 2001–2016. (**C**) Clinical outcome of human cases of YF in Brazil during 2001–2016. All data was obtained from Sistema de Informação de Agravos de Notificação (SINAN), and epidemiological reports of the Brazilian Ministry of Health.

The recent re-emergence of YFV showed that the majority of the population affected by YF (82.8% during 2017–2018) were male. This population is in an economically active age range [12] and is composed of residents of rural areas, probably due to work activities and proximity to forest sites, factors that contribute to the exposure of these individuals to YFV vectors [3,40,41]. Considering the data from Sistema de Informação de Agravos de Notificação (SINAN), an increase in the number of YF cases affecting male individuals has been observed since 2001 (Figure 3). The higher prevalence in males brings economic losses to their families and the region, as men are more likely to perform most of the activities in the field. Moreover, it is important to highlight that deforestation is a factor that can increase the risk of YFV spread to urban environments, raising opportunities for human exposure to fragmented forest areas with the occurrence of YFV sylvatic cycles [11,43].

The 2016–2019 YF epidemic also brought additional economic impacts for health authorities and for the public health in general. It is already known that the emerging infectious diseases (EID) cause significant impacts such as high costs associated with response plans, surveillance, and preventive actions [44]. Certainly, the re-emergence of YFV in Brazil caused a great burden for the public health services, since hundreds of Intensive Care Unit (ICU) beds were needed, in addition to expenses related to the monitoring of patients in the different clinical stages of the disease, and the laboratory diagnosis of human and epizootics cases.

In this sense, one of the major challenges faced during the 2016–2019 epidemic was the establishment of standard protocols for clinical management of patients, which culminated in the implementation of the national catastrophe plan implemented by the government for the 2014 World Cup, at least by Minas Gerais state (the epicenter of outbreaks). In many hospitals, there was a change in patient management areas, an increase in the number of ICU beds and the hiring of healthcare professionals to serve the affected population. In addition, there was a need to create a transportation system for patients from rural areas to large urban centers, where reference hospitals and higher acuity care were located (Serviço de Infectologia do Hospital Eduardo de Menezes, reference of Minas Gerais state for Yellow Fever, personal communication). This decision was taken on an urgent basis, taking into account the rapid spread of YF and the worsening of the patients’ clinical conditions. In fact, YF can present a broad clinical spectrum in humans, including asymptomatic infection, mild illness and severe disease; however, much is still unknown about the pathogenesis of this disease [3,5]. Considering this last outbreak in Brazil, a great advance in the disease’s understanding has been reported and new clinical findings and outcomes have been described in the literature. Recent studies have reported the occurrence of late-relapsing and persistent hepatitis after YF [45,46] and other clinical findings were the occurrence of pancreatitis and progressive severe metabolic acidosis in severe cases of YF and manifestations in the central nervous system [47,48].

Certainly, the clinical management of severe forms constituted a major challenge since YF leads to liver failure with rapid evolution, compromises other vital organs and leads to a cytokine storm that culminates in plasma leakage and shock, which implies the need for intensive supportive therapies and treatments. Regarding the clinical management of patients with severe YF, Ho and colleagues showed that some measures such as the use of anticonvulsant drugs, routine use of intravenous proton pump inhibitors, aggressive early haemodialysis and plasma exchange were beneficial during YF treatment [47]. Additionally, studies related to understanding the predictors of mortality in patients with severe forms of YF affected during previous epidemic periods have been shown to be valuable. Hence, factors such as increasing age, male gender, higher neutrophil and leukocyte count, higher aspartate transaminase (AST) and alanine aminotransferase (ALT), bilirubin, and creatinine, prolonged prothrombin time, and higher viral load are significantly used as predictors of mortality of YF disease [49].

Furthermore, the recent YF epidemic also raised possibilities for increasing the knowledge related to the clinical field and antiviral research, with recent studies showing that the repurposing of clinically approved drugs can represent a quick alternative to discover new antivirals for public health emergencies. Freitas et al. [50] demonstrated that YFV is susceptible, in vitro and in vivo, to sofosbuvir, a clinically approved drug against Hepatitis C virus (HCV). In addition, Mendes et al. [51] demonstrated a reduction in blood viremia and an improvement in clinical course with sofosbuvir treatment.

The management of vaccination in the affected population can also be considered a complex challenge in the YF epidemic scenario. In order to protect the largest possible portion of population against the disease, the Sistema Único de Saúde (SUS) distributed 45.1 million doses of the YFV vaccine in 2017 and 23.8 million doses in 2018 [52]. Currently, the North, Midwest, Southeast, and South regions of Brazil are considered areas with vaccination recommendation (ACRV), however, in the Northeast region, the vaccination has been recommended only for Bahia and Maranhão states, and in some municipalities of Piauí, Sergipe, and Alagoas states [39]. Vaccination campaigns against YF also aim to prevent expansion of viral circulation, which is also associated with the movement of people [40]. The 17DD vaccine produced by the Institute of Technology in Immunobiologicals (Bio-Manguinhos) of the Oswaldo Cruz Foundation (Fiocruz) leads to 98% protection [2,11].

The target public for which the vaccine is indicated are individuals from 9 months to 59 years of age who reside or travel to areas with vaccination recommendation [2,34]. In the current campaign, the vaccine is being used in a standard dose (0.5 mL), but during the YF epidemic period, the dose fractionation strategy was adopted for some states, with the fractional dose (0.1 mL) corresponding to a fifth (1/5) of a standard dose [11,34,52,53]. This type of strategy is approved during emergency situations and has already been recommended by the World Health Organization (WHO) and used in outbreaks such as during the one that occurred in 2015–2016 in the African continent [52,53,54,55,56]. As the viral dissemination was favored by the vaccine shortage, with insufficient doses for the entire population, the vaccine fractionation strategy was an effective tool. In addition, strategies to control the *Ae aegypti* vector, which contribute to preventing viral spread and the resurgence of YFV, have little emphasis on the continent [57]. However, after emergency situations it is necessary to re-vaccinate the population with the full dose; the long-term protection provided by fractional doses in varied populations and epidemiological contexts is unknown [58]. In this sense, the study by Costa-Rocha and colleagues which evaluated the duration of humoral and cellular immunity after administration of reduced doses of the 17DD-Yellow Fever vaccine provided evidence to support the regular use of dose sparing strategies for YF vaccine in adults [59].

Currently, the WHO recommends one life-time dose of the YF vaccine; however, this is controversial for two reasons: the level of neutralizing antibodies drops years after vaccination and cases of YF infection have occurred in previously vaccinated individuals [40,60,61,62,63]. Even though YF vaccine is highly immunogenic and able to induce a robust antibody response and a strong and polyfunctional cellular immune response, recent studies demonstrated the importance of booster doses to ensure a long-term persistence of memory components in response to 17DD YF vaccine [60,61,62,64]. These recent findings suggest that in YF endemic areas, at least an additional dose of the vaccine should be administered after the first immunization, in order to avoid the reduction in neutralizing antibodies titers below the protective levels [60,61,62]. During the recent epidemic in Brazil, the priority was to vaccinate the largest portion of the population possible; however, it is recommended that after this emergency period the population receives a full dose of 17DD vaccine, reinforcing the idea that at least two doses are necessary [58,60,62,65].

With the re-emergence of YFV significantly affecting the Southeastern region of Brazil, and the consequent concern about the beginning of an urban cycle, there was an intensification of vaccination campaigns mainly in large urban centers [34,52]. The dislocation of cases, previously restricted to rural areas, to metropolitan regions, as recorded mostly in 2018, was of great concern and alert to epidemiological surveillance systems. However, the low vaccination coverage against YF in Brazil is a problem that has persisted for decades [66]. In view of this, the current challenge is to achieve vaccination coverage of at least 95% in all Brazilian territory.

A recent study conducted by Stoffella-Dutra and colleagues revealed that, respectively, 25.8% and 26.5% of the rural and urban populations living in Serro region (state of Minas Gerais) did not present neutralizing antibodies against YFV [67]. Furthermore, 10 individuals from the same area that presented their vaccination card with proven vaccination against YFV tested negative for the presence of neutralizing antibodies. Considering the recent epidemic and the risks of YF re-urbanization, this finding raises questions about the real burden of YFV infections, in which the disease could be underestimated. Although the vaccination coverage in that region has improved [68], there are still a high number of individuals lacking any neutralizing antibodies response against YFV, which can increase the vulnerability of the populations, as well as the to the occurrence of new outbreaks or even epidemics [67].

Other studies performed in Brazil also highlighted the absence of neutralizing antibodies against YFV in individuals from rural and urban areas [69,70]. However, different from the results reported by Stoffella-Dutra and colleagues, few individuals tested negative. These findings draw attention to the fact that some areas in the state of Minas Gerais can present a high proportion of individuals with absence of neutralizing antibodies against YFV, which could potentially be a factor related to the recent 2016–2019 YF epidemic, reinforcing this state as the epicenter of the outbreaks [40].

The presence of individuals lacking a neutralizing antibodies response against YFV in endemic areas for YF disease also reinforces the importance of active epidemiological surveillance and continued vaccination campaigns aiming to reach at least ≥95% coverage. This coverage is necessary because according to the WHO, a vaccine coverage of at least 80% would be important to prevent and control new outbreaks [71]. Indeed, learning about the current seroprevalence in regions under risk of YF disease can add valuable information that could help to assist national and international health authorities in the development of future vaccination strategies.

The 2016–2019 YF epidemic in Brazil was the most impactful in the past decades and several factors, including the failure of entomological and epizootic surveillance systems, low vaccination coverage in several regions of the country, and population migration, have potentially contributed to the recent epidemic scenario. In this context, issues related to vector control and NHP surveillance are also challenges to be covered.

Effective surveillance of the vector population is essential for implementing control strategies, as well as contributing to highlighting potential sources of transmission and potential new outbreaks, not only for YF, but also of other recurrent arboviruses in Brazil such as Zika, Dengue and Chikungunya [72]. Considering the plasticity of vectors involved in the YF transmission cycle and the existence of an urban vector widely domesticated in the country, it would be important that the PAHO Entomology and Vector Control Action Plan [73] become implemented, maintaining an active surveillance not only in Brazil but for all affected countries in America.

Similar to vector control strategies, NHP epizootics surveillance still need to be better established and optimized in all regions of Brazil for a greater control effectiveness, especially in those areas with higher risks of enzootic cycles. Hence, epizootics surveillance needs to be carried out in a sufficient time frame, capable of providing effective control for viral dispersion. Furthermore, information about illness and death in the NHP, obtained by the Epizootics Surveillance Program of Brazil and local health authorities, also fails to report and disseminate data.

The effective diagnosis of YF human cases is also another critical point. The delay in reporting the laboratory test results slowed down the processes of clinical intervention and surveillance. Apart from this, another issue that compromises better understanding of epidemics is the classification of cases into “cases under investigation”, which makes it difficult to understand the true dimension of the epidemic event due to the delay observed for resolution in cases discarded or confirmed as YFV infection or YF disease. This delay directly influences the possible actions to control and fight against the disease.

Considering the old classification of yellow fever’s endemic area in the country, the Brazilian coast and the Southeastern region were considered YF free areas, however, with the re-emergence of YFV in 2016, this scenario can no longer be considered (Figure 2). There are factors that may be related to viral dislocation for the East and South regions of the country, among them the ecological changes (fragmentation of habitats, climate changes) and the patterns of human behavior, which may have contributed to the increased densities of vectors and NHP, and their consequent proximity to humans [5,11]. Political and social factors are also relevant in the complex recent re-emergence scenario of YF in Brazil, such as failure of political commitment and strategies to achieve satisfactory vaccine coverage and monitor population immunity in areas at risk for YF transmission. Besides poor basic sanitation in several regions of the country, which can contribute to the proliferation of vectors, anthropic environmental changes, such as the advancement of agriculture and peri-urban growth and insufficient health and surveillance policies, can contribute to the poor detection and control of outbreak situations [11,71]. Furthermore, Faria and colleagues estimated that virus lineages moved, on average, 4.25 km/day during the last outbreak. This velocity on vectors movement also reflected YFV lineage movement within the enzootic cycle and not the movement of asymptomatic infected humans. These findings also corroborate the fact that NHPs are not likely to carry the virus over long distances [11,40], different from infected humans and vector species that can help with viral dislocation, reaching greater distances [11,35,74].

Recent phylogenetic studies have shown that YFV strains circulating during the 2016–2019 epidemic presented a high identity with the South American genotype I, previously described circulating in the Amazon region [40,41,74,75,76]. Further analysis revealed that the strain 1E were responsible for the recent epidemic, which was not associated with previous outbreaks already described in the Southeast region in 2000 and in 2008 [40,74,75,76,77,78,79,80]. Genomic analyses of YFV samples from this latest epidemic showed that the YFV lineage responsible for the 2016–2019 outbreak originated from Midwest region, spreading to Minas Gerais state at least two times, and reaching two distinct routes in the Southeastern region of Brazil [80]. The most affected states until 2018 were Minas Gerais, São Paulo, Rio de Janeiro and Espírito Santo. During 2019, São Paulo continued to report cases, with others being also reported in the states of Paraná and Santa Catarina [12,39].

## 4. Progress and Perspectives

**Figure 4:**
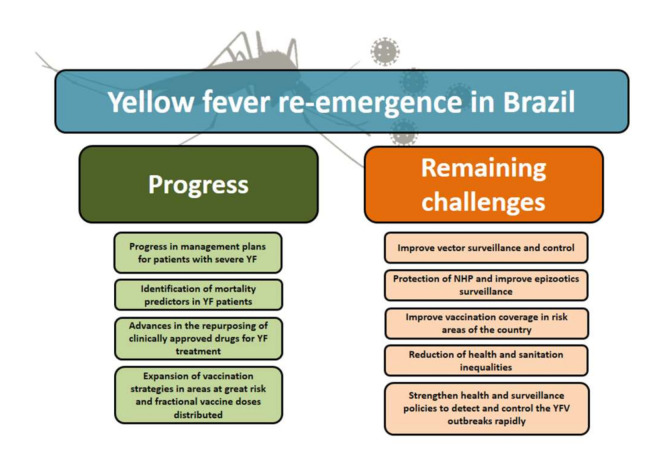
An overview of the YF re-emergence in Brazil. The flowchart highlights some progress that can be recognized during the 2016–2019 epidemic, and the challenges that still need to be covered to contribute to the surveillance and control of YF in Brazil.

The surveillance and control of vector populations are actions that must be constant in the YF context, especially in outbreak situations. The prevalence and distribution of vectors species are important key indicators of the risk of the occurrence of an urban cycle in certain regions and the necessity of direct control measures towards areas that need to be prioritized [71,81]. In this sense, it is important to think about a combination of mosquito control strategies that should include policies to improve basic sanitation in large cities and other strategies for direct control of the mosquito population, in addition to strategies such as the introduction of genetically modified or biologically manipulated mosquitoes. An example of this is the global initiative World Mosquito Program (WMP), which uses the symbiotic bacterium *Wolbachia* as a biocontrol tool to reduce the transmission of mosquito-borne diseases [82]. In Brazil, the *Wolbachia* method is coordinated by the Oswaldo Cruz Foundation (Fiocruz), under the guidance of the Ministry of Health, and the first releases of *Aedes aegypti* mosquitoes carrying *Wolbachia* began in 2014 in Rio de Janeiro, and the project is expected to reach 2.38 million people by 2023 [82]. Scientific evidence has demonstrated the ability of *Wolbachia* to reduce the transmission of Dengue, Zika, Chikungunya, and Yellow Fever viruses by the *Aedes aegypti* mosquitoes [83,84,85,86]. For YF control, this approach helps to prevent the onset of urban cycles of the disease [86]. In addition to all the challenges here exposed, Figure 4 represents an overview in terms of progress and remaining challenges related to the latest YF outbreak in Brazil.

Finally, and equally important is to know that the identification of epizootics provides an early warning of viral circulation and helps prevent YF human cases. Thus, the adoption of strategies for the protection of NHPs is fundamental not only for the conservation of species, but also due to the importance of these animals as sentinel hosts of the disease. In the context of sylvatic YF re-emergence experienced in Brazil, all confirmed human cases had as a probable site of infection (LPI) areas with previous occurrence of epizootics in NHP (Figure 2) [12,40,87].

Furthermore, it is important to note that the vector species distribution is related to YFV epidemiology, and, in this context, even more expressive epidemics can be caused when infected individuals are inserted in densely populated areas with the urban vector *A. aegypti* [9,14]. This species is the main vector for many flaviviruses, and it is widely distributed in the urban centers of the Southeastern region, and, due to the active circulation of YFV in this region, the threat of an urban cycle has proved to be concrete [13,15,74].

Vector competence analyses showed that the anthropophilic mosquitoes *A. aegypti* and *A. albopictus* are highly susceptible to be infected and transmit YFV, in addition to the wild vectors *Haemagogus leucocelaenus* and *Sabethes albiprivus* [13]. It is important to highlight the presence of *A. albopictus* in Brazil, as it has spread throughout the country since 1980. Experimental studies suggested that YFV has the potential to adapt to *A. albopictus*, which is an opportunistic species, since it can move between urban and peri-urban habitats [13,88]. In addition, little is known about other vectors (especially the *Culicidae*) and their potential role in YFV transmission. Most of the work related to vector competence to sylvatic virus was performed a long time ago, and their results may not reflect the real-world data since the strain of virus and mosquitoes have been evolving and changing over the years. Taking into account the proximity of YFV circulation to the Atlantic coast and to large urban centers, it is worth noting that even poorly competent vectors can pose a threat in the transmission of pathogens if some factors such as high vector density, high human-biting rate and high survival rates are present. Thus, factors that may have favored the re-emergence of YFV in Brazil such as the presence of susceptible human populations, favorable climatic conditions, and the circulation of infected humans and NHPs are undeniable [11,13,35].

## 5. Conclusions

The emergence of zoonotic viruses is an important challenge for global public health, now more than ever in our connected world due of international travel and trade in which highly contagious diseases can quickly spread. Understanding the ecological gaps related to the EID and the impact of social changes on the control and prevention of possible epidemics is a challenge that requires international cooperation. The complex YF eco-epidemiology in Brazil is a case study to demonstrate the challenge for public health agencies and policy makers to effectively control and monitor disease. The fact that YF is a zoonosis and cannot be eradicated is an aggravating factor, especially considering the increased proximity of human populations to viral circulation areas. However, for a disease that presents high mortality in severe cases, the large population of unvaccinated and susceptible individuals may favor viral spread and re-emergence events in densely populated areas. Moreover, with viral circulation now present in the Atlantic Forest, close to large metropolitan areas in the Southeastern region, the risk of YF re-urbanization is highly concerning for human health and potential international viral spread. The occurrence of enzootic cycles makes arboviruses, especially YFV, a recurrent issue. From this point of view, it is important to emphasize the need to use efficient tools to prevent introductions of YFV into the urban cycle, such as the combination of efficient vector control strategies and large-scale vaccination campaigns. Hence, there is an urgent need to strengthen the Brazilian health systems in order to improve decision-making for control, response and prevention of future YF and other EID outbreaks.
